# Significantly Lower Anti-*Leishmania* IgG Responses in Sudanese versus Indian Visceral Leishmaniasis

**DOI:** 10.1371/journal.pntd.0002675

**Published:** 2014-02-20

**Authors:** Tapan Bhattacharyya, Duncan E. Bowes, Sayda El-Safi, Shyam Sundar, Andrew K. Falconar, Om Prakash Singh, Rajiv Kumar, Osman Ahmed, Marleen Boelaert, Michael A. Miles

**Affiliations:** 1 Faculty of Infectious and Tropical Diseases, London School of Hygiene and Tropical Medicine, London, United Kingdom; 2 Faculty of Medicine, University of Khartoum, Khartoum, Sudan; 3 Institute of Medical Sciences, Banaras Hindu University, Varanasi, Uttar Pradesh, India; 4 Departamento de Medicina, Universidad del Norte, Barranquilla, Colombia; 5 Immunology and Infection Laboratory, Queensland Institute of Medical Research, Herston, Queensland, Australia; 6 Department of Laboratory Medicine, Karolinska Insitutet, Stockholm, Sweden; 7 Department of Public Health, Institute of Tropical Medicine, Antwerp, Belgium; University of Texas Medical Branch, United States of America

## Abstract

**Background:**

Visceral leishmaniasis (VL), a widely distributed systemic disease caused by infection with the *Leishmania donovani* complex (*L. donovani* and *L. infantum*), is almost always fatal if symptomatic and untreated. A rapid point-of-care diagnostic test for anti-*Leishmania* antibodies, the rK39-immunochromatographic test (rK39-ICT), has high sensitivity and specificity in South Asia but is less sensitive in East Africa. One of the underlying reasons may be continent-specific molecular diversity in the rK39 antigen within the *L. donovani* complex. However, a second reason may be differences in specific IgG anti-*Leishmania* levels in patients from different geographical regions, either due to variable antigenicity or immunological response.

**Methodology/Principal Findings:**

We determined IgG titres of Indian and Sudanese VL patients against whole cell lysates of Indian and Sudanese *L. donovani* strains. Indian VL patients had significantly higher IgG titres against both *L. donovani* strains compared to Sudanese VL patients (p<0.0001). Mean reciprocal log_10_ 50% end-point titres (1/log_10_t_50_) were i) 3.80 and 3.88 for Indian plasma and ii) 2.13 and 2.09 for Sudanese plasma against Indian and Sudanese antigen respectively (p<0.0001). Overall, the Indian VL patients therefore showed a 46.8–61.7 -fold higher mean ELISA titre than the Sudanese VL patients. The higher IgG titres occurred in children (<16 years old) and adults of either sex from India (mean 1/log_10_t_50_: 3.60–4.15) versus Sudan (mean 1/log_10_t_50_: 1.88–2.54). The greatest difference in IgG responses was between male Indian and Sudanese VL patients of ≥ 16 years old (mean 1/log_10_t_50_: 4.15 versus 1.99 = 144-fold (p<0.0001).

**Conclusions/Significance:**

Anti-*Leishmania* IgG responses among VL patients in Sudan were significantly lower than in India; this may be due to chronic malnutrition with Zn^2+^ deficiency, or variable antigenicity and capacity to generate IgG responses to *Leishmania* antigens. Such differential anti-*Leishmania* IgG levels may contribute to lower sensitivity of the rK39-ICT in East Africa.

## Author Summary

Visceral leishmaniasis (VL) is a systemic disease with highest prevalence in South Asia, East Africa, and Brazil. VL is caused by protozoan parasites of the *Leishmania donovani* complex, transmitted to humans when an infected sandfly takes a bloodmeal. Within the human host, the parasites replicate within cells, particularly of bone marrow and spleen. Without effective treatment, symptomatic VL is usually fatal. Correct treatment depends on accurate diagnosis, which is by detection of parasites or specific antibodies. The rK39 rapid diagnostic test for antibody is highly sensitive in South Asia but less so in East Africa, for poorly understood reasons. Here, we have directly compared the anti-*Leishmania* antibody response in groups of VL patients from India and Sudan. We found a strikingly higher anti-*Leishmania* antibody response in Indian compared to Sudanese patients, which was also seen when further analysed by age and sex of the patients. Thus in addition to parasite factors, we have shown that difference in antibody levels may contribute to the lower sensitivity of antibody-based diagnosis for VL in Sudan.

## Introduction

The great majority of the estimated 200,000 to 400,000 annual new cases of visceral leishmaniasis (VL) occurs in six countries, with India having the highest estimated incidence in the world (146,700 to 282,800/year), Sudan having the highest in Africa (15,700 to 30,300/year) and Brazil having the highest in the Americas (4,200 to 6,300/year) [1]. In South Asia and East Africa, VL is caused by the kinetoplastid protozoan *Leishmania donovani*, transmitted by the sandfly vectors *Phlebotomus argentipes* in South Asia and *P. orientalis* and *P. martini* in East Africa. Following inoculation into the human host, the parasite disseminates through the lymphatic and vascular systems. Some infected individuals remain asymptomatic, but full-blown symptomatic VL with bone marrow infiltration and hepatosplenomegaly is almost always fatal if untreated [2].

The demonstration of *L. donovani* amastigotes in lymph node, spleen or bone marrow tissue smears is the definitive diagnostic method for infection, however due to the invasive nature and the operational difficulties associated with these procedures, serological assays have been developed. Serological (anti-*Leishmania* antibody) tests include the enzyme-linked immunosorbent assay (ELISA), indirect fluorescent antibody test (IFAT) and the direct agglutination test (DAT) [3], [4]. However, these antibody detection tests remain positive for several months to years after drug treatment and cure and therefore cannot readily diagnose relapse; such tests can also be positive in asymptomatic individuals living in endemic areas and exposed to *L. donovani* infection yet with no history of VL or subsequent progression to VL.

The lateral-flow rapid diagnostic ‘point-of-care’ immunochromatographic test (ICT) format based on the rK39 antigen derived from a Brazilian isolate of *Leishmania infantum* (historically known as *L. chagasi*) [5] has demonstrated high levels of sensitivity in South Asia but is less effective in East Africa for diagnosis of VL [6], [7]. A new ICT, based on the synthetic gene rK28, has recently been developed to overcome these limitations [8], [9], and is currently under evaluation. Underlying explanatory factors for the different levels of rK39 diagnostic success observed across geographical regions may be molecular divergence between East African *L. donovani* kinesin gene homologues and the Brazilian *L. infantum* (*L. chagasi*)-derived rK39 sequence [10], and/or may be due to quantitative differences in the IgG titres generated against the rK39 antigen between South Asian and East African VL-endemic populations. Here, we compare anti-*L. donovani* IgG titres in cases of active VL in children and adults of each sex from India and Sudan against whole cell lysates of *L. donovani* strains from both countries. We find striking differences between the anti-*Leishmania* IgG titres of the two human populations.

## Methods

### Ethics statement

In India, comparative serology was approved by the Ethics Committee of the Banaras Hindu University, Varanasi, India. In Sudan the protocols were approved by the Ethical Research Committee, Faculty of Medicine, University of Khartoum and the National Health Research Ethics Committee, Federal Ministry of Health. Written informed consent was obtained from all adult subjects included in the study, or from the parents or guardians of individuals less than 18 years of age. This research was also covered by the London School of Hygiene and Tropical Medicine Ethics Committee approval of the EC NIDIAG project.

### Study populations

Sudan: plasma samples were obtained upon clinical presentation from active cases of VL in the Gedaref region in eastern Sudan between July and September 2011. Patients were diagnosed as positive for VL by a combination of bone marrow aspiration, lymph node aspiration, or serology. India: plasma samples were obtained upon clinical presentation from active cases of VL in Bihar state, north-eastern India after 2009. Active VL patients were diagnosed by identification of parasitologically-positive splenic aspirates. All samples in Sudan were transported by continuous cold chain with samples maintained at 4°C, to the research laboratory at Suba University Hospital and there stored at −80°C; similarly all samples in India were cold chain transported at 4°C to the laboratory at Banaras Hindu University and stored at −80°C.

### Antigen preparation

Strains of *L. donovani* originating from Sudan (MHOM/SD/97/LEM3458) and India (MHOM/IN/80/DD8) were cultured in αMEM (Sigma, UK) supplemented as described [11]. Mid-to-late log phase cultures were washed three times in phosphate-buffered saline (PBS), followed by three cycles of flash-freezing in liquid nitrogen and thawing in a cold water bath. Subsequently, cells were subjected to three 30 sec 12-micron sonication cycles on ice at 30 second intervals using a Soniprep 150 sonicator (MSE, UK). Sonicates were centrifuged at 12000×*g* for 1 min, and the supernatant used as antigen. Protein concentrations in these lysates were determined using the BCA Protein Assay kit (Fisher Scientific, Loughborough, UK).

### Anti-*Leishmania donovani* ELISA

**Figure 1:**
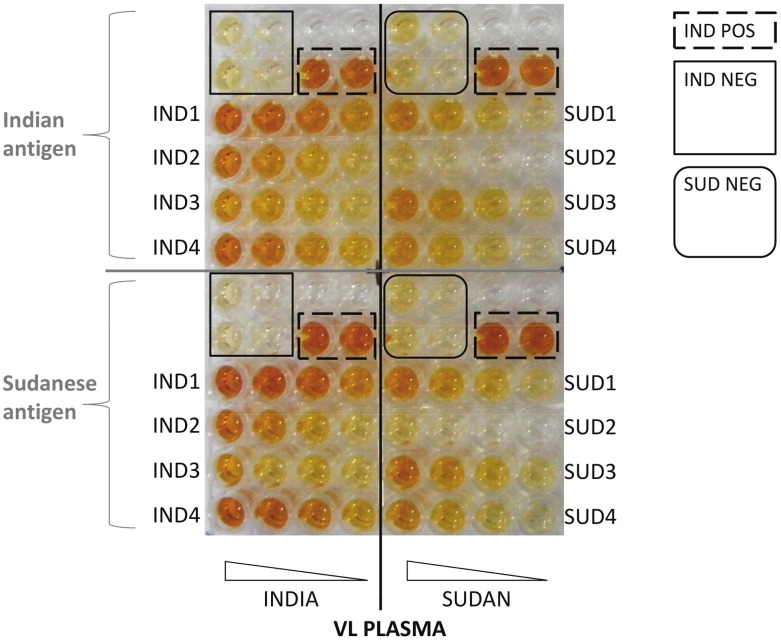
Design of assay for comparative ELISA serology. Serial four-fold dilutions of plasma samples from Indian and Sudanese VL patients were reacted with whole cell lysates of *L. donovani* strains isolated from each country. Identically formatted plates were run in all cases. Unlabelled wells were not used.

**Figure 2:**
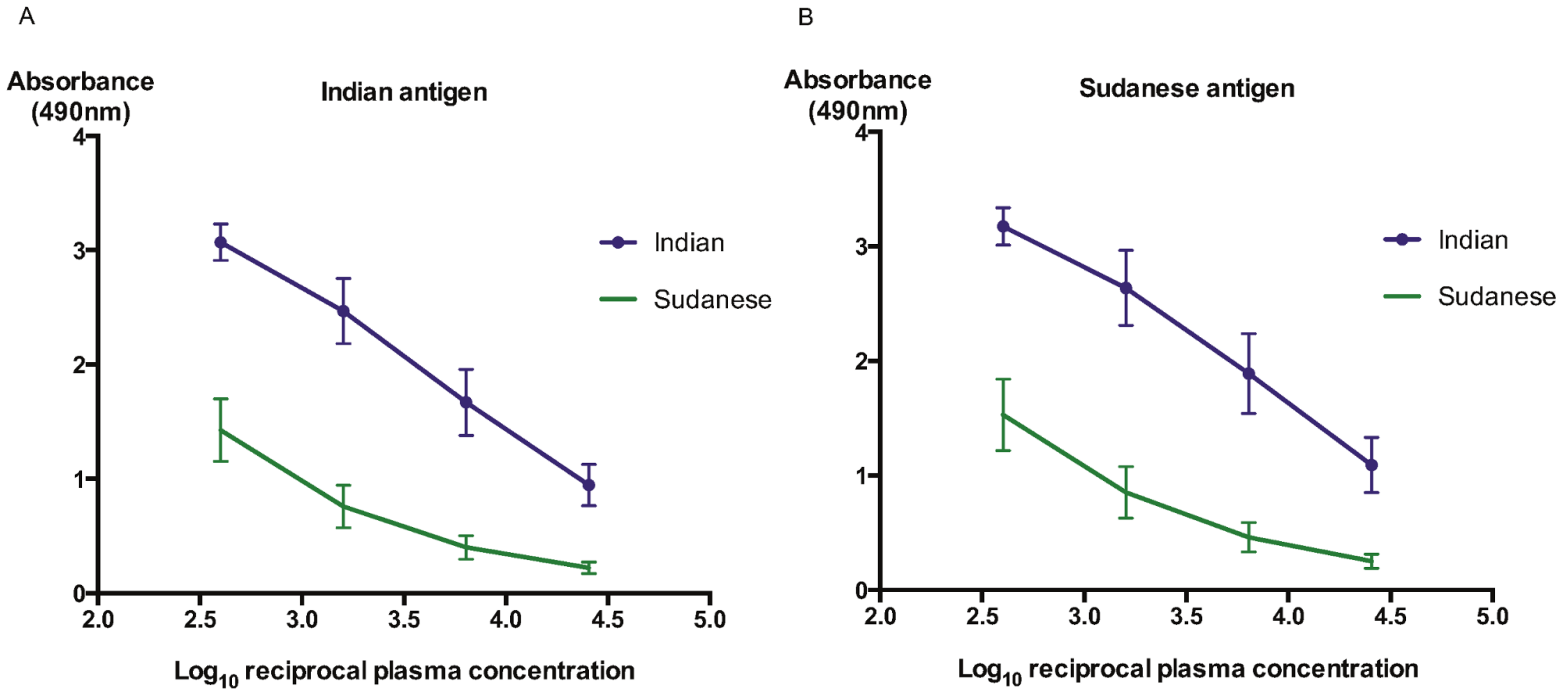
IgG anti-*Leishmania* responses are higher in Indian than Sudanese VL patients. The mean IgG responses are shown with 95% CI, for 36 Indian (purple line with nodes) and 36 Sudanese (green line) patients with active VL, against lysates of *L. donovani* strains isolated from [A] India or [B] Sudan. Comparative mean 1/log_10_t_50_ IgG titres and fold-differences are shown in Table 2. Statistical p values of <0.0001 were obtained for both the Indian and Sudanese antigens.

The indirect *L. donovani* ELISA was performed using relatively low antigen concentrations (0.2 µg/well) established by prior ELISA checkerboard titrations (not shown), with the intention of increasing ability to discriminate between patients generating high and low IgG antibody responses. Lysates of the *L. donovani* DD8 (Indian) and LEM3458 (Sudanese) strains, diluted to 2 µg/ml in 35 mM NaHCO_3_/15 mM NaCO_3_ buffer (pH 9.6), were separately added at 100 µl/well to 48 wells (A to H 1–6 or A to H 7–12) (see Figure 1) of Immulon 4HB ELISA plates (VWR, Lutterworth, UK) and incubated overnight at 4°C. After washing the plates three times using PBS containing 0.05% (vol/vol) Tween 20 (Sigma, Gillingham, UK) (PBST), they were blocked using 200 µl/well PBST containing 2% skimmed milk powder (Premier International Foods, Spalding, UK) (PBSTM) at 37°C for 2 hr. After washing three times with PBST, serial four-fold 1∶400 to 1∶25,600 dilutions of VL plasma samples (Table 1, Figure 1) (100 µl/well) in PBSTM were added to the plates and incubated at 37°C for 1 hr. Plasma samples from the two endemic countries were assayed on the same plate against antigens of both Indian and Sudanese strains and were matched for sex and age groups as shown in Figure 1. Following six PBST washes, a 1∶5,000 dilution of peroxidase-labelled goat anti-human IgG (H+L) (Jackson ImmunoResearch, West Grove, USA) prepared in PBSTM was added at 100 µl/well and the plates incubated at 37°C for 1 hr. Following six PBST washes, 50 mM phosphate/citrate buffer (pH 5.0) containing 2 mM *o*-phenylenediamine HCl and 0.007% (vol/vol) H_2_O_2_ (Sigma, UK) was added at 100 µl/well and incubated in the dark at room temperature for 15 minutes. The substrate reactions were then stopped by the addition of 2 M H_2_SO_4_ (50 µl/well) and the ELISA plates were read at 490 nm (Spectra Max 190, Molecular Devices, Sunnyvale, USA). Coefficients of variation (mean positive control readings (n = 4)/standard deviation of positive control x 100) at dilutions of 1/400 and 1/1600 were calculated from simultaneous duplicate plates for both Indian and Sudanese sera (most coefficients of variation were less than 1%). Reference positive plasma controls were also used on every plate (Figure 1). Samples that gave coefficients of variation above 20% were repeated. Representative plasma samples from Indian and Sudanese endemic healthy controls and from cases of active VL were used as inter-assay controls. These plasma samples were chosen in preliminary assays as having median responses against the lysate obtained from the *L. donovani* strain isolated from the corresponding region (data not shown). To maximize the chances of identifying differences in the IgG responses against the two different *L. donovani* strains (Figure 2) and between Indian and Sudanese VL patients of each sex and age group (≥ or <16 years old), the highest mean absorbance (Abs_max_) value was determined for each data set, from which the Abs_max_/2 was determined for calculating the mean reciprocal log_10_ 50% end-point (1/log_10_t_50_) ELISA titres. This methodology has been recommended for comparisons between high, low and non-classical IgG responses of acute and convalescent phase samples from both individuals and patient groups infected with another pathogen [12], [13]. This approach is more accurate than determining ELISA titres as either: i) single end-point values located on the very trailing slope of the ELISA sigmoid curve (e.g. values that are 2 standard deviations above the mean obtained using negative control sera) or ii) the mean absorbance values obtained using single serum dilutions (e.g. 1/100) [12]. In the latter case, the limited use of absorbance values results in a low dynamic range of data for comparisons [12].

**Table 1:** Indian and Sudanese plasma study populations used in comparative serology against *Leishmania donovani* antigens.

Endemic region	Sex	Age (years)	n	Mean age in years (range; standard deviation)
India (n = 36)	Male	≥16	10	33.4 (16–70; 17.45)
		<16	8	12.8 (10–15; 1.67)
	Female	≥16	8	30.5 (17–52; 12.22)
		<16	10	12.8 (9–15; 2.20)
Sudan (n = 36)	Male	≥16	10	22.2 (16–43; 9.17)
		<16	8	9.8 (4–15; 4.1)
	Female	≥16	7	35.6 (25–60; 12.35)
		<16	11	8.1 (1–15; 4.55)
Total n = 72				

### Statistical analyses

Two sided independent sample t-tests were used to analyse the data (SPSS INC. Armonk, NY: IBM Corp). Normality was assessed using a three tiered approach. Shapiro-Wilk tests were conducted first with subsequent evaluation of the data through visual assessment and by calculating a z-score for skewness (Z_Skewness_ = Skewness-0/SE _Skewness_) as proposed by Ghasemi and Zahediasl [14]. In the event of a violation of the assumption of homogeneity of variance (homscedasticity), SPSS calculated a corrected p value. Significance was set at the 5% level.

## Results

Table 1 summarises the age and sex compositions of the Indian and Sudanese VL patients who provided plasma for comparisons of serological responses between the two endemic regions.

### Comparison of Indian and Sudanese IgG isotype responses

**Figure 3:**
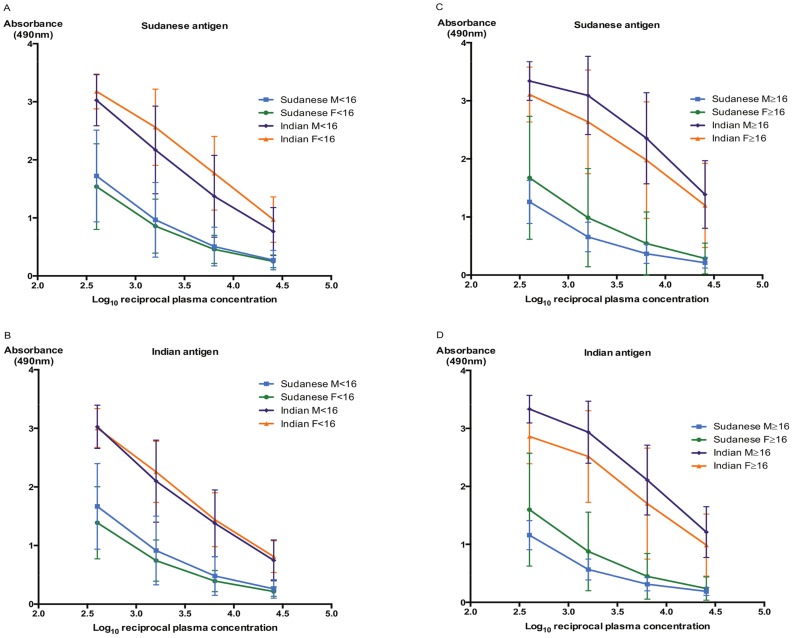
IgG anti-*Leishmania* responses are higher in Indian VL regardless of age, sex or antigen source. The mean IgG responses and 95% CI are shown for Indian (purple and orange lines) and Sudanese (blue and green lines) active VL patients against lysates of *L. donovani* strains isolated from Sudan [A & C] or India [B & D]. Comparative mean 1/log_10_t_50_ IgG titres and fold-differences are given in Table 2.

The mean overall Sudanese (n = 36) and Indian (n = 36) active VL patients' IgG responses, at each plasma dilution, against lysates of *L. donovani* strains isolated from each endemic area, with 95% confidence intervals (CIs) are shown in Figure 2. In a few instances, the unforeseen low Sudanese titres required minor extrapolation of curves to determine specific 1/log_10_50% end-point titres but since homoscedasticity was obtained in each case a correction factor was not required. Similar high mean maximum absorbance (Abs_max_) values were obtained for the Indian VL patients against both the Indian and Sudanese *L. donovani* strains. However the Indian VL patients showed significantly higher IgG titres against both the Sudanese (mean 1/log_10_t_50_: 3.88) and Indian (mean 1/log_10_t_50_: 3.80) *L. donovani* strains than the Sudanese VL patients (mean 1/log_10_t_50_: 2.09 and 2.13 respectively) (Figure 2A and B, Table 2; two-sided independent sample t-tests for both *L. donovani* strains: p<0.0001). Thus, overall the Indian VL patients generated 46.8–61.7-fold higher IgG titres than the Sudanese patients against the *L. donovani* strains (Table 2). We further assessed whether there were differences in anti-*L. donovani* IgG titres generated by the male and female Indian and Sudanese VL patients of less than or greater than 16 years old against both *L. donovani* strains. We again used the mean highest absorbance (Abs_max_) value for each data set (Figure 3A to D) to maximize the identification of differences in the mean IgG responses, from which the Abs_max_/2 value was determined for interpolating the mean 1/log_10_t_50_ IgG ELISA titres. We observed that the male VL patients of ≥16 years old from India showed higher Abs_max_ values against both the Sudanese (Abs_max_ 3.34) and Indian (Abs_max_ 3.33) antigens than the female Indian VL patients of the same age group (Abs_max_ 3.11 and 2.99, respectively) (Figure 3C and D). This difference was less in the male and female Indian VL patients of <16 years old (Figure 3A and B). Importantly, the Sudanese male and female children (<16 years old) and adults all generated lower mean IgG ELISA titres against both *L. donovani* strains than the corresponding Indian VL patient sex/age groups (Figure 3A to D). These differences ranged from 11.5 to 144-fold (Table 2).

**Table 2:** Mean reciprocal ELISA titres and fold differences of Indian and Sudanese VL patients by age, sex and antigen source.

Mean reciprocal 50% end-point titre								
				(1/log_10_t_50_) interpolated from Abs_max_/2				
Figure	Sex	Age	Antigen source	Indian plasma	Sudanese plasma	Fold difference	p value (95% CI)	p value (95% CI)
						(Indian-Sudanese)	Sex: M or F	Both Sexes
2A	Both	All	Sudan	3.88	2.09	61.7	-	p<0.0001 (1.35–2.24)
2B	Both	All	India	3.80	2.13	46.8	-	p<0.0001 (1.29–2.06)
3A	M	<16	Sudan	3.63	2.52	12.9	p<0.007 (0.356–1.87)	p<0.0001 (0.929–2.21)
	F	<16	Sudan	3.78	1.88	79.4	p<0.001 (0.898–2.91)	
3B	M	<16	India	3.60	2.54	11.5	p<0.004 (0.385–1.73)	p<0.0001 (0.90–2.03)
	F	<16	India	3.69	1.92	58.9	p<0.001 (0.883–2.66)	
3C	M	≥16	Sudan	4.15	1.99	144	p<0.0001 (1.45–2.87)	p<0.0001 (1.38–2.67)
	F	≥16	Sudan	3.95	2.09	72.4	p<0.02 (0.388–3.33)	
3D	M	≥16	India	4.03	2.02	102	p<0.0001 (1.53–2.48)	p<0.0001 (1.36–2.40)
	F	≥16	India	3.84	2.13	51.3	p<0.008 (0.539–2.89)	

The Sudanese male VL patients less than 16 years old showed the lowest fold-differences compared to the Indian male VL patients of the same age group (12.9-fold and 11.5-fold against Sudanese and Indian antigen respectively). In contrast, the Sudanese male VL patients older than 16 years showed the highest fold-difference with the Indian male patients of the same age group (144-fold).

## Discussion

According to a recent report from the World Health Organisation, among the research priorities for human diseases caused by infection with kinetoplastid protozoa is research on diagnostics for case detection and characterisation [15]. For many years, the rK39 antigen has been the only rapid diagnostic ICT in a lateral flow system that is readily applicable for field diagnosis of VL and that can be used with minimal training with no other equipment or reagent. Despite high levels of sensitivity in South Asia, the rK39 ICT has shown lower efficacy in East Africa, for reasons that are not fully understood. The recent rK28 ICT, based on 2×39 amino acid repeats of a Sudanese *L. donovani*-derived kinesin homologue of rK39, flanked by HASPB sequences, has been developed in an attempt to overcome the differential sensitivity of rK39. Reduced efficacy in East Africa has also been reported for a diagnostic test using another *L. donovani* antigen, rKE16 [7].

Different sensitivities of the rK39 ICT in South Asia and East Africa may be explained by molecular divergence in diagnostic antigen sequences of geographically disparate *L. donovani* strains and/or reflect the different levels of overall IgG anti-*Leishmania* response between human populations in VL endemic areas. We have assessed the first of these factors, and demonstrated substantial and regional specific antigen diversity [10], an observation subsequently reported by others [16].

Differential serological responses among different ethnic groups within the same geographical region have been reported previously, for both bacterial and protozoal pathogens, although generally without understanding of the genetic/biological explanations. Higher responses were found against: *Helicobacter pylori* in non-Japanese Brazilians than Japanese Brazilians [17]; *Streptococcus pyogenes* superantigen in Polynesian than New Zealand Europeans [18]; *Plasmodium falciparum* in Fulani than sympatric ethnic groups in Burkina Faso [19]; *P. falciparum* in Austro-Asiatic than Tibeto-Burman groups in north-east India [20]. Jensen et al [21] reported comparatively higher anti-*Plasmodium* titres in subjects from Flores (Indonesia) than in counterparts from southern Sudan. In the UK, higher IgG levels were found in South Asian patients compared to European counterparts in the context of cardiovascular disease [22].

Here, we used comparative analysis of serological responses, as applied elsewhere [12], [13] to assess accurately differences in IgG titres between VL patient cohorts from Bihar (India) and Gedaref (Sudan). We have clearly shown that active VL patients from India generated significantly higher anti-*L. donovani* IgG responses against whole cell lysates of both Indian and Sudanese parasite strains than active VL patients from Sudan. This may contribute to the reduced sensitivity of the commercial rK39 ICT assay with Sudanese versus Indian VL patients. We used soluble antigens from *Leishmania* whole cell lysates in the comparative ELISAs. On western blots human VL serum antibodies recognise multiple antigens in such lysate preparations (data not shown). It is therefore likely that Sudanese patients with active VL had reduced IgG titres against several *L. donovani* promastigote antigens; we have not yet determined whether the response to particular antigens is depleted. Thus, it may be challenging to identify *L. donovani* antigens that provide adequately high sensitivity and specificity for East Africa. Whilst it would be of interest to extend these comparisons to include IFAT, results from ELISA and IFAT generally accord, and we therefore anticipate that the two tests will give compatible data [23]. Detection of antigens in urine may provide an alternative, non invasive approach, possibly giving prognostic information [24], [25]. In the same multi-centre trial comparing rK39 dipstick sensitivity and specificity across South Asia and East Africa, Boelaert et al. [6] also compared the KAtex® test, which detects a *Leishmania* carbohydrate antigen in urine [26], [27]. Sensitivities in both regions were described as moderate to very low, with the lowest in India and Nepal, although higher sensitivities have been recently reported from Bangladeshi studies [28]–[30].

We have not yet assessed antibody levels in patients with post-kala azar dermal leishmaniasis (PKDL). This is of interest because PKDL is a sequela of VL that displays markedly different epidemiological features between regions, in Sudan occurring at a higher frequency (>50%) and much sooner after VL than in India (<10%) [31]. In Sudan and India the plasma was collected at time of clinical presentation of VL and no obvious differences in symptoms between the two cohorts were observed; further comparisons would benefit from more precise grading of clinical severity.

Whilst the active VL patient numbers used in our analysis were relatively low, Indian males ≥16 years old with active VL generated higher IgG responses than females of the same age group, whereas Sudanese male VL patients ≥16 years old generated lower IgG responses than females of the same age group. These trends were not apparent in Indian or Sudanese patients <16 years old. Differences in infection between males and females in late teenage years and in adults may be attributable to increased susceptibility of young and adult men [32]; testosterone increases *L. donovani* infection in macrophages [33]. In Sudan VL is a disease of extreme poverty with high incidence associated with chronic malnutrition, displacement of populations and lack of health care [1], [34]–[39]. Particular ethnic groups, possibly with greater genetic susceptibility, may be more affected within the ethnically mixed Sudanese population [40], [41].

Malnutrition is known to reduce both human innate and acquired immune responses against parasitic diseases such as VL [42], [43]. Experimental models of protein, zinc and iron malnutrition have shown reduced immune responses in *L. infantum* infected mice and increased early visceralisation [44], [45]. Of these, even mild zinc deficiency has been extensively reported to: i) reduce T- and B -cells in the blood and lymphoid tissues of humans, non-human primates and other animals, ii) dramatically reduce both T- and B- cell numbers in the bone marrow by >75%, iii) dramatically reduced B-cell IgG production, particularly to T-dependent antigens, by 90% and iv) reduce both T- and B- cell proliferation, resulting in depletion through apoptosis [46]. More specifically, zinc deficiency in mice was shown to block the development of bone-marrow pre-B and immature B-cells, resulting in reduced B lymphocytes in the spleen; pre-natal zinc deficiency in monkeys and mice reduced lymphocyte numbers and IgG concentrations and produced long-term suppression of IgM, IgA and IgG. Zinc deficiency in mice also resulted in reduced B-cell responses to recall antigens with which they had previously been inoculated, thereby suggesting that T- and B- cell depletion resulted in impaired immunological memory [46]. Thus, malnutrition with zinc deficiency, even if relatively mild and previously encountered may play an important role in the significantly lower IgG titres of the Sudanese patients.

Human interleukin-2 (IL-2) and signalling through its receptor (IL-2R) play a critical role in both T- and B-cell proliferation and induce the expression of the *IL-2Rα* gene in B cells, thereby increasing B-cell responses to IL-2, antibody heavy and light chain expression and antibody secretion [47]. Thus, single nucleotide polymorphisms in the genes encoding IL-2 and its receptor (IL-2Rα or IL-2Rβ) chains significantly affect human IgG titres in response to both inactivated and live attenuated vaccines (e.g. the live attenuated measles-mumps-rubella (MMR) vaccine) [48], [49]. While there have not been similar studies performed on Sudanese populations, VL patients in eastern Sudan had a significant association with two SNPs in the *IL-2Rβ* gene, one of which encoded a non-conserved amino acid substitution (G245R) in the proximal membrane domain that was very likely to affect IL-2 signalling [50]. Thus, these SNPs and others located in the genes that affect IL-2 expression, function and responses may have contributed to the reduced IgG responses of Sudanese VL patients observed in our study.

Human leukocyte antigen (HLA) polymorphisms in the genes encoding the MHC class II (HLA-DR, -DP and -DQ) molecules are known to have significant effects on IgG responses to vaccines [48], infectious diseases, cancer, asthma and autoimmune diseases [51]–[53]. Early studies did not however find a significant association of HLA class II polymorphisms in VL patients in either Tunisia or Brazil [54], [55]. By contrast, in a more recent study performed using VL patients' samples from Brazil and India, *HLA-DRB1* and *HLA-DQA1* were the only genes associated with VL risk in both populations [56] but comparisons of anti-*L. donovani* IgG titres between these groups or the control groups were not performed. Since no similar HLA studies have been performed on Sudanese VL patients, it is presently not yet known whether polymorphisms in these genes may have contributed to the significantly lower IgG titres observed in the Sudanese versus Indian VL patients found in our study.

Thus, the significant differences in the anti-*L. donovani* IgG titres found here between the Indian and Sudanese VL patients in this study may result from a combination of environmental and genetic factors, in which malnutrition with Zn^2+^ deficiency, differences in IL-2 responses due to polymorphisms in *IL-2* and its receptor (*IL-2Rα* and *IL-2Rβ*) genes and possibly *HLA-DRB1* and *HLA-DQA1* genes are to date the most likely candidates.

We have presented here a direct ELISA comparison of serological responses between VL patients from different endemic regions (Sudan and India), and have shown a clear difference in the levels of IgG anti-*Leishmania* antibodies. Comparative serological responses could also be extended to samples from Brazilian cases of VL to investigate their contribution to reported lower sensitivity with rK39-based ICTs [7], despite this diagnostic antigen being derived from a Brazilian *L. infantum* (*L. chagasi*), dispersed from Iberian Europe [57]. Our data suggest that the relative levels of ability to mount a humoral response against *Leishmania* antigen between the different human populations may be a significant contributory factor in the differing levels of sensitivity reported for rapid diagnostic tests applied in both regions, and argue for the design and development of a test more suited to East Africa, guided by comparative genomics [10].
